# Thioether-Linked Liquid Crystal Trimers: Odd–Even Effects of Spacers and the Influence of Thioether Bonds on Phase Behavior

**DOI:** 10.3390/ma15051709

**Published:** 2022-02-24

**Authors:** Yuki Arakawa, Kenta Komatsu, Yuko Ishida, Takuma Shiba, Hideto Tsuji

**Affiliations:** Department of Applied Chemistry and Life Science, Graduate School of Engineering, Toyohashi University of Technology, 1-1 Hibarigaoka, Tempaku-cho, Toyohashi 441-8580, Japan; komatsu.kenta.qr@tut.jp (K.K.); ishida.yuko.rm@tut.jp (Y.I.); shiba.takuma.wt@tut.jp (T.S.); ht003@edu.tut.ac.jp (H.T.)

**Keywords:** liquid crystal trimer, odd-even effect, thioether, twist-bend nematic phase, smectic phase

## Abstract

We report the synthesis, phase-transition behavior, and mesophase structures of the first homologous series of thioether-linked liquid crystal (LC) trimers, 4,4′-bis[ω-(4-cyanobiphenyl-4′-ylthio)alkoxy]biphenyls (CBS*n*OBO*n*SCB with a wide range of spacer carbon numbers, *n* = 3–11). All CBS*n*OBO*n*SCB homologs exhibited LC phases. Interestingly, even-*n* and odd-*n* homologs showed monotropic layered smectic A (SmA) and pseudo-layered twist-bend nematic (N_TB_) phases, respectively, below a nematic (N) phase. This alternate formation, which depends on spacer chain parity, is attributed to different average molecular shapes, which are associated with the relative orientations of the biphenyl moieties: linear and bent shapes for even-*n* and odd-*n* homologs, respectively. In addition, X-ray diffraction analysis indicated a strong cybotactic N phase tendency, with a triply intercalated structure. The phase-transition behavior and LC phase structures of thioether-linked CBS*n*OBO*n*SCB were compared with those of the all-ether-linked classic LC trimers CBO*n*OBO*n*OCB. Overall, thioether linkages endowed CBS*n*OBO*n*SCB with a monotropic LC tendency and lowered phase-transition temperatures, compared to those of CBO*n*OBO*n*OCB, for the same *n*. This is attributed to enhanced flexibility and bending (less molecular anisotropy) of the molecules, caused by the greater bond flexibility and smaller inner bond angles of the C–S–C bonds, compared to those of the C–O–C bonds.

## 1. Introduction

**Figure 1:**
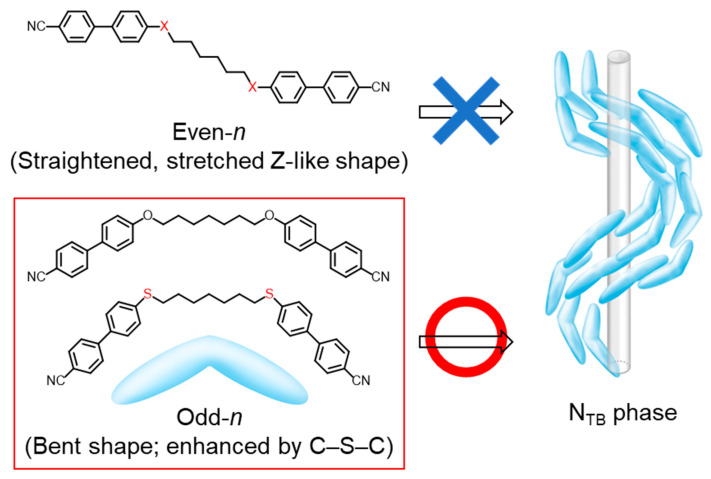
Molecular geometries (shapes) imposed by spacer parity of semi-flexible spacers, and schematic model of the N_TB_ phase formed by bent molecules. The model of the N_TB_ phase was reproduced from ref. [70] with permission from Wiley.

Liquid crystal (LC) dimers, trimers, and higher oligomers have been widely investigated [1]. They consist of more than two rigid mesogenic groups linked linearly via flexible alkylene spacers, as shown in Figure 1. A fascinating feature of LC oligomers is the odd–even effect of the number of spacer-atoms on various LC properties, associated with the relative orientation of rigid mesogenic groups, or different molecular geometries. In simple dimers, even-number spacers roughly direct the mesogenic groups to a parallel alignment, forming a relatively straightened stretched Z-like shape, whereas odd-number spacers form bent shapes, as shown in Figure 1. This contrast generates odd–even oscillations in various physical properties associated with the orientational order parameter [1,2,3,4,5,6].

The average geometrical differences influence the types of LC phases induced [7,8]. The discovery of a heliconical twist-bend nematic (N_TB_) phase for bent LC dimers with odd-number spacers has provided a strong impetus to explore oligomeric LC materials in the last decade [9,10,11,12]. The N_TB_ phase has nanoscopic heliconical structures with pitches ranging from several nanometers to tens of nanometers [13,14,15]. Interestingly, chiral helical structures develop from achiral bent molecules, with the degeneracy of right- and left-handed helical structures. These helical structures cause the N_TB_ phase to apparently involve layered smectic (Sm) phase-like behavior, which is pseudo-layered in nature [16,17,18,19,20]. Physical properties of twist-bend nematogens have been extensively studied for basic scientific reasons as well as potential applications [21,22,23,24,25,26,27,28,29,30,31,32]. However, the phase identification and nanoscopic structures of the N_TB_ phase require further investigation [33,34,35].

**Figure 2:**
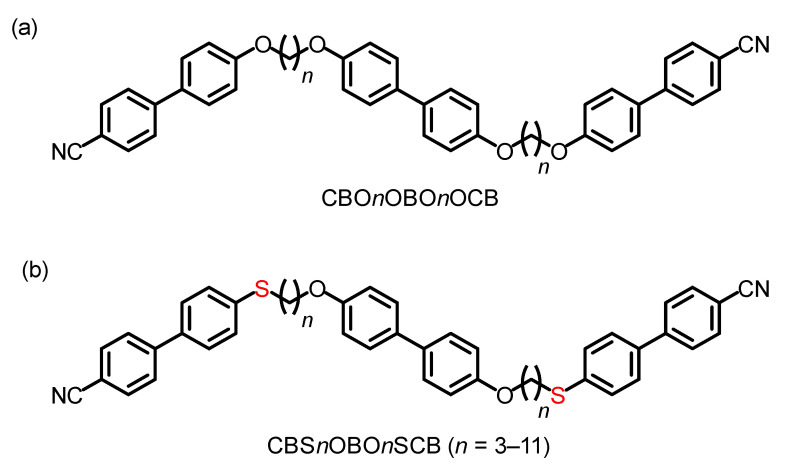
Molecular structures of (**a**) all-ether-linked biphenyl-based 4,4′-bis[ω-(4-cyanobiphenyl-4′-yloxy)alkoxy]biphenyl homologs (CBO*n*OBO*n*OCB, *n* = 4–11) (previously reported) [58,59,60,61], and (**b**) their outer thioether-linked homologs (CBS*n*OBO*n*SCB, *n* = 3–11) (the present study), in which *n* = 7 and 9 have been previously reported [62,63].

Intensive studies have confirmed that many LC dimers [36,37,38,39,40,41,42,43,44,45,46,47,48,49] and some oligomers [50,51,52,53,54], polymers [55], and bent-core molecules [56,57] exhibit the N_TB_ phase. Higher oligomers that have more mesogenic units are less likely to form an N_TB_ phase [55]. In contrast to the many reports on dimer homologs containing the same mesogenic cores and different alkylene spacers, only one homologous series of LC trimers has been studied as twist-bend nematogenic higher oligomer homologs [58]. This is a homologous series of the all-ether-linked biphenyl-based LC trimers, 4,4′-bis[ω-(4-cyanobiphenyl-4′-yloxy)alkoxy]biphenyls (CBO*n*OBO*n*OCB with spacer carbon numbers, *n* = 4–11, where B, CB, and O denote central 4,4′-linked biphenyl, bilateral cyanobiphenyl groups, and ether linkages, respectively), which are the classic LC trimer homologs [59,60,61]. It has been reported that the CBO*n*OBO*n*OCB homologs alternately form layered smectic A (SmA) and pseudo-layered N_TB_ phases for even-*n* and odd-*n*, respectively [58]. Furthermore, their outer thioether-linked analogs, CBS*n*OBO*n*SCB with odd *n* = 7 and 9 (Figure 2a) also exhibit the N_TB_ phase below the N phase [62,63]. Flexible C–S–C bonds have smaller bond angles (~103°) than ethers (C–O–C; ~118°). This generally reduces the ability of calamitic molecules to form LCs, by providing greater conformational and steric bulkiness [62,63,64,65,66,67]. In contrast, the smaller bond angle of the flexible C–S–C bond synergistically contributes to N_TB_ phase induction and stabilization [62,63,68,69,70,71,72,73,74] by enhancing the bent shape and flexibility of LC oligomers with odd-*n* spacers, as shown in Figure 1. This is also the case for the higher chalcogen selenoether C–Se–C bond [75]. To date, other CBS*n*OBO*n*SCB homologs including even-*n* have not been synthesized, so the influences of thioether linkages on the phase-transition behavior, its odd–even effects, and LC phase structures of a homologous series of LC trimers have not been studied.

In this context, we developed a homologous series of the thioether-linked CBS*n*OBO*n*SCB trimers possessing a wide variety of odd and even numbers of carbon atoms in the alkylene spacers (*n* = 3–11), of which *n* = 7 and 9 have been previously reported [62,63]. Their phase transitions and structures were investigated by polarized optical microscopy (POM), differential scanning calorimetry (DSC), and X-ray diffractometry (XRD), and compared with those of the corresponding all-ether-linked trimers, CBO*n*OBO*n*OCB [58].

## 2. Materials and Methods

Synthetic schemes and procedures for CBS*n*OBO*n*SCB are described in the Supplementary Material. Molecular structures were determined by ^1^H and ^13^C nuclear magnetic resonance (NMR) spectroscopy, using a JNM-ECX500 (500 MHz for ^1^H, and 125 MHz for ^13^C NMR) or a JNM-ESC400 (400 MHz for ^1^H, and 100 MHz for ^13^C NMR) instrument (JEOL, Ltd., Tokyo, Japan). ^1^H and ^13^C NMR data are provided in the Supplementary Material, excluding ^13^C NMR data of even-*n* homologs. Their spectra could not be obtained because they had poor solubility similar to ether-linked CBO*n*OBO*n*OCB trimers [58]. Phase identification was determined by POM using an Olympus polarizing microscope (BX50, Tokyo, Japan), equipped with a Linkam temperature controller (LK-600PM, Surrey, UK). POM observations were carried out for the samples that were placed between non-treated thin glass cells. The obtained POM microphotographs are shown in the main text and Supplementary Material. Phase-transition temperatures for crystal (Cr)–N or Cr–isotropic (I) phase transitions upon heating (melting point: *T*_m_), crystallization upon cooling (*T*_Cr_), N–I phase transitions upon heating and cooling (*T*_NI_ and *T*_IN_, respectively), monotropic N–SmA and N–N_NTB_ phase transitions upon cooling (*T*_NS_ and *T*_NNTB_, respectively), and associated entropy changes (Δ*S*) scaled by the gas constant (*R*) on *T*_m_, *T*_Cr_, *T*_NI_, and *T*_IN_ (Δ*S*_m_/*R*, Δ*S*_Cr_/*R*, Δ*S*_NI_/*R,* and Δ*S*_IN_/*R*, respectively), were determined by DSC, using a DSC-60 Plus instrument (Shimadzu, Kyoto, Japan), which was calibrated using indium. DSC measurements were conducted upon heating-to-cooling-to-heating cycles at a rate of 10 °C min^−1^ under a flow of nitrogen gas. Samples (3–4 mg) weighed accurately in aluminum pans were used for the DSC measurements. The obtained DSC curves are shown in the Supplementary Material, except for those of the previously reported CBS*n*OBO*n*SCB (*n* = 7 and 9) [62,63]. In addition, the phase-transition temperatures for the elusive monotropic LC phases were determined by POM. The N phases of CBS9OBO9SCB and CBO9OBO9OCB were analyzed by XRD measurements, using a D8 DISCOVER diffractometer, with a Cu Kα radiation source, equipped with a Vantec-500 detector (Bruker, Billerica, MA, USA). Capillary glass tubes with 1.5 mm diameter (WJM-Glass Müller GmbH, Berlin, Germany), in which the material was kept, were sandwiched between permanent magnets in a homemade folder. Measurements were carried out at different temperatures upon cooling from each I phase, to remove the thermal history of the materials.

## 3. Results and Discussion

### 3.1. Phase-Transition Behavior of CBSnOBOnSCB

**Figure 3:**
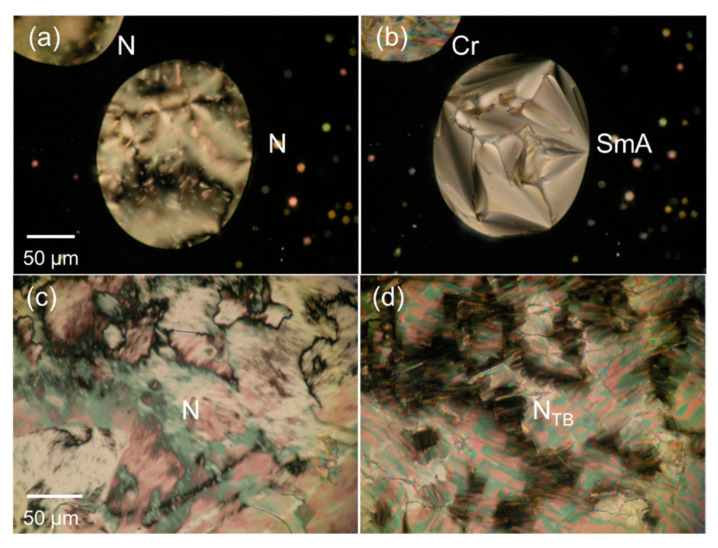
POM images of CBS6OBO6SCB [(**a**,**b**)] and CBS11OBO11SCB [(**c**,**d**)]: (**a**) Marble and schlieren textures (N phase) at 210 °C; (**b**) Fan-shaped and focal conic textures (SmA phase) with a Cr region at 178 °C; (**c**) Marble and schlieren textures (N phase) at 130 °C; (**d**) Blocky texture (N_TB_ phase) at 119 °C in a non-treated glass cell.

The phase-transition results obtained upon first heating and cooling CBS*n*OBO*n*SCB are listed in Table 1. The different *T*_m_ and phase transitions, which could be associated with the existence of crystal polymorphs, were observed depending on the thermal courses and conditions. For simplicity, these are not discussed in this paper. All CBS*n*OBO*n*SCB trimers exhibited the conventional N phases. More specifically, the N phases of *n* = 4, 7–9, and 11 were enantiotropic, while those of *n* = 3, 5, 6, and 10 were monotropic. Notably, even-*n* and odd-*n* CBS*n*OBO*n*SCB trimers (for *n* = 5–11) exhibited monotropic layered Sm and pseudo-layered N_TB_ phases, respectively, below the N phase temperature, as reported for the all-ether-linked CBO*n*OBO*n*OCB trimers [58]. This is a part of the odd–even effect on the phase-transition behavior of LC trimers. Considering that the N_TB_ phase is induced only by bent molecules, this alternate formation of Sm and N_TB_ phases could be ascribed to the dependence of the average molecular shapes on the parity of *n* for the LC trimer (straightened and bent shapes for even-*n* and odd-*n* homologs, respectively). Even-*n* homologs (*n* = 6, 8, and 10) exhibited fan-shaped textures of layered Sm phases. These Sm textures were observed in the supercooled N phase domains or droplets after crystallization of major parts, as shown in Figure 3a,b, Figures S4 and S5, for *n* = 6, 8, and 10, respectively. Therefore, these monotropic Sm phases were unsuitable for further characterization using XRD. Considering the homeotropic dark textures reported for the Sm phases of all-ether-linked CBO*n*OBO*n*OCB homologs with even-*n* [58,61], those of thioether-linked CBS*n*OBO*n*SCB could be characterized as orthogonal SmA phases. POM microphotographs of the N_TB_ phase of CBS11OBO11SCB are shown in Figure 3c,d, where blocky and focal conic-like textures were observed in a non-treated glass cell. These textures are characteristic of the N_TB_ phase, and are caused by the undulations of the pseudo-periodic layers of the helical structures [14,17,19]. The N–N_TB_ phase transitions for CBS5OBO5SCB were observed in small, supercooled N domains and droplets by POM (Figure S3). The results for *n* = 7 and 9 have been previously reported [62,63]. In this study, however, the above-mentioned optical textural changes, which indicate the presence of monotropic SmA or N_TB_ phases, were not observed for the shortest even/odd CBS*n*OBO*n*SCB homologs (*n* = 3 and 4), which exhibited only the N phase, as shown in Figures S1 and S2.

**Figure 4:**
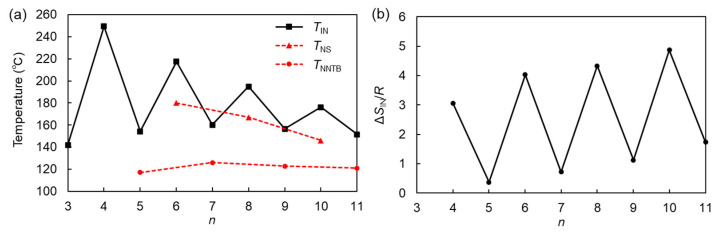
(**a**) Phase-transition temperatures upon cooling: *T*_IN_ (squares), *T*_NS_ (triangles), and *T*_NNTB_ (circles), and (**b**) Δ*S*_IN_/*R* upon cooling, as a function of *n*, for the CBS*n*OBO*n*SCB trimers.

To analyze the spacer-length dependency of phase-transition behavior, *T*_NS_, *T*_NNTB_, *T*_IN_, and Δ*S*_IN_/*R* upon cooling are plotted as a function of *n* for the CBS*n*OBO*n*SCB trimers (Figure 4). Because of the monotropic LC natures for the N (for *n* = 3, 5, 6, and 10) and SmA or N_TB_ phases (for *n* = 5–11), the cooling data are discussed here. As shown in Figure 4, CBS*n*OBO*n*SCB homologs exhibited clear odd–even oscillations for *T*_IN_ and Δ*S*_IN_/*R*, with much higher values for even-*n* than for odd-*n*. Such oscillation, depending on the parity of *n*, is also observed for *T*_m_ upon heating. These odd–even effects on phase transitions are generally attributed to the different average molecular shapes imposed by spacer parity. Even-*n* and odd-*n* make the molecular shapes of LC trimers linear and bent, respectively. It is typically observed that, with increasing *n*, although the odd–even oscillation magnitude for *T*_IN_ decreased (or attenuated), the odd–even oscillation for Δ*S*_IN_/*R* was unattenuated. Interestingly, the *T*_NS_ and *T*_NNTB_ of LC trimers visually resembled odd–even oscillations for successive *n* values, as shown in Figure 4a, despite the different types of LC phases involved. With increasing *n*, the *T*_NS_ for even-*n* decreased sharply, whereas the *T*_NNTB_ for odd-*n* slightly increased. Similar trends have been reported for CBO*n*OBO*n*OCB [58]. Even-*n* homologs formed Sm phases, which are ascribable to their roughly linear shapes and the lengthening of their alkylene spacers that caused a significant separation of parallel oriented biphenyls. The significant decline in *T*_NS_ with increasing *n* could be attributed to the C–S–C bond linkage causing high conformational and steric bulkiness of the two rotatable terminal cyanobiphenyls. In contrast, the N_TB_ phase was driven only by bent molecular shapes. The molecular anisotropy of bent LC oligomers, which have an odd number of spacers, increased with the number of spacers. Therefore, the *T*_NNTB_ increased upon increasing *n*, and vice versa. The *T*_NNTB_ values of the biphenyl-based LC trimers (>110 °C) are clearly higher than those of usual biphenyl-based dimers (<100 °C). Increased intermolecular interactions, e.g., higher oligomers [58,76] and extended π-conjugation [70], lead to increasing *T*_NNTB_. 

### 3.2. Comparison of CBSnOBOnSCB and CBOnOBOnOCB

#### 3.2.1. Phase-Transition Behavior

**Figure 5:**
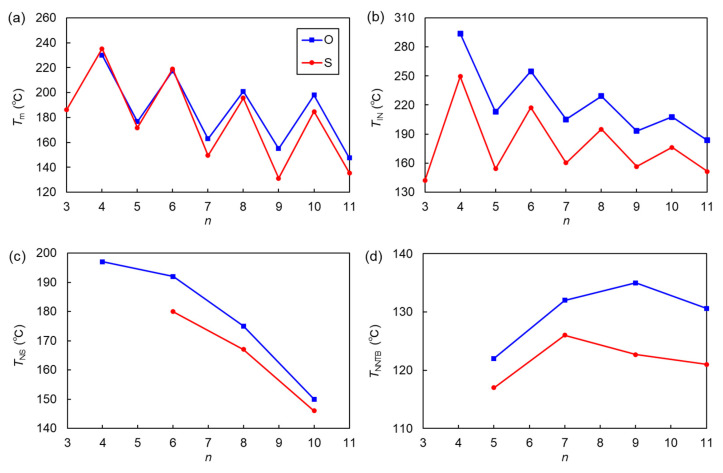

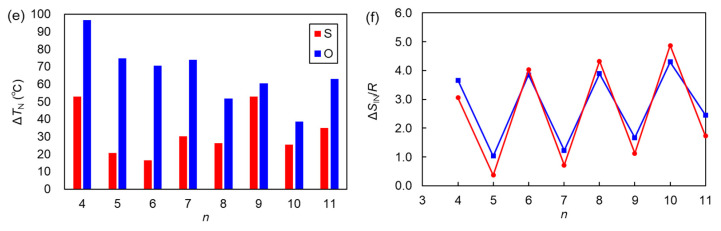
Comparisons of (**a**) *T*_m_, (**b**) *T*_IN_, (**c**) *T*_NS_, (**d**) *T*_NNTB_, (**e**) Δ*T*_N_, and (**f**) Δ*S*_IN_/*R*, as a function of *n*, for the CBS*n*OBO*n*SCB and CBO*n*OBO*n*OCB trimer homologs.

The phase-transition behavior of thioether-linked CBS*n*OBO*n*SCB and all-ether-linked CBO*n*OBO*n*OCB homologs were compared. *T*_m_, *T*_IN_, *T*_NS_, *T*_NNTB_, N phase temperature range upon cooling (Δ*T*_N_), and Δ*S*_IN_/*R* of both the homologs are plotted as a function of *n* (Figure 5). The data for CBO*n*OBO*n*OCB is quoted from the literature [58].

As described above, the thioether-linked CBS*n*OBO*n*SCB (*n* = 4, 7–9, and 11) exhibited the enantiotropic N phase, whereas the homologs with *n* = 3, 5, 6, and 10 exhibited the monotropic N phase. This differs from the behavior of all-ether-linked CBO*n*OBO*n*OCB homologs, which exhibit enantiotropic N phases for all tested *n* values (4–12) [58,59,60,61]. Thus, the replacement of outer ether linkages with thioether reduced the ability of the trimer homologs to form LCs [63], which is similar to the behavior of thioether-containing calamitic LCs [64,65,66,67]. This is primarily ascribed to the large conformational flexibility, steric bulkiness, and lower molecular anisotropy (larger molecular width) that the C–S–C bond imparts because it is more flexible, shorter, and has a smaller angle (~100°) than the ether C–O–C bond.

Figure 5a shows that the *T*_m_ values of both homologs oscillated significantly with the parity of *n*. The *T*_m_ values of thioether-linked CBS*n*OBO*n*SCB homologs became lower than those of CBO*n*OBO*n*OCB, with increasing *n*. As shown in Figure 5b–d, the thioether-linked CBS*n*OBO*n*SCB homologs exhibited lower *T*_IN_, *T*_NS_, and *T*_NNTB_ values than their CBO*n*OBO*n*OCB counterparts. Additionally, Δ*T*_N_ was smaller (narrower) for thioether-linked CBS*n*OBO*n*SCB than for CBO*n*OBO*n*OCB, as shown in Figure 5e, primarily because of the lower *T*_IN_ (or *T*_NI_) of CBS*n*OBO*n*SCB. In addition to the tendency to form monotropic LCs, the lower phase-transition temperatures and consequent narrower temperature LC phases for thioether-linked LC trimers are primarily ascribed to the greater conformational flexibility, steric bulkiness, and the low molecular anisotropy afforded by flexible C−S−C bonds with smaller inner angles than C−O−C bonds. For bent LC dimers, the thioether linkage causes the LC phases to be significantly supercooled and vitrified. This is a consequence of the synergy between the enhanced bent shape of the entire molecule and the flexibility of the C−S−C bond, which prevents crystallization [68,70,71,72,73,74]. However, such supercooling effects on LC phases were not observed for the thioether-linked trimers in this study, which all crystallized. Notably, Figure 5f reveals that the Δ*S*_IN_/*R* values of CBS*n*OBO*n*SCB were lower and slightly higher than those of CBO*n*OBO*n*OCB for odd-*n* and even-*n*, respectively. Therefore, the replacement of the outer ether with thioether caused a greater odd–even oscillation in Δ*S*_IN_/*R*. This trend is similar to the difference between methylene- and ether-linked cyanobiphenyl-based dimer homologs (CB*n*CB and CBO*n*OCB, respectively), in which the methylene linkages lead to the greater odd–even oscillation than the ether linkages [3]. More specifically, when the spacer has odd-*n*, Δ*S*_IN_/*R* is lower for a methylene-linked CB*n*CB dimer than for an ether-linked CBO*n*OCB counterpart. This is ascribed to more bent geometry or greater biaxiality for CB*n*CB, which could be the case for the present trimers that more bent CBS*n*OBO*n*SCB show lower Δ*S*_IN_/*R* than CBO*n*OBO*n*OCB. In contrast, when the spacer has even-*n*, Δ*S*_IN_/*R* is higher for CB*n*CB than for CBO*n*OCB, which is associated with the higher order parameters for CB*n*CB than for CBO*n*OCB [3]. This consideration may possibly be linked to the present thioether-linked CBS*n*OBO*n*SCB trimers for even-*n* to be higher Δ*S*_IN_/*R* than CBO*n*OBO*n*OCB.

#### 3.2.2. Mesophase Structures

XRD measurements were performed for thioether-linked CBS9OBO9SCB and all-ether-linked CBO9OBO9OCB, which contain the same nonane spacers. For both these materials, prior crystallization upon cooling, unfortunately, prevented the determination of the diffraction patterns of the monotropic SmA and N_TB_ phases. Therefore, only the data for the upper N phases are described.

**Figure 6:**
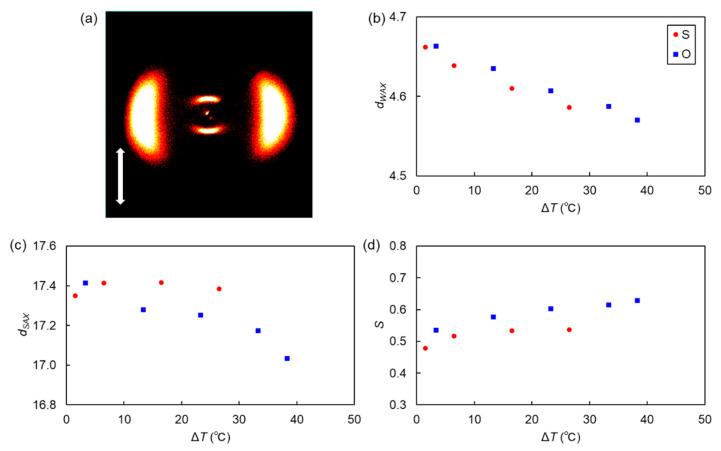
(**a**) The 2D-XRD pattern of the N phase (155 °C) of CBS9OBO9SCB in a magnetic field denoted by a meridional arrow, and (**b**) *d*_WAX_, (**c**) *d*_SAX_, and (**d**) *S* values, as a function of Δ*T*, for the CBS9OBO9SCB and CBO9OBO9OCB trimer homologs.

Two-dimensional (2D) XRD patterns obtained for the N phases of both trimers showed two symmetric arc-shaped diffractions on the equatorial line in the wide-angle region, as shown in Figure 6a and Figure S13. These indicate the average orientation of the molecular long-axis along the magnetic field directions, denoted by a meridional arrow in Figure 6a. The wide-angle diffractions are associated with average lateral intermolecular correlations. The *d*-spacing values of the wide-angle diffractions (*d*_WAX_) of both trimers were estimated using the Bragg equation and are plotted in Figure 6b as a function of shifted temperature, Δ*T* = *T*_IN_ − *T*, where *T* refers to measured temperature upon cooling. The *d*_WAX_ values were in the range of 4.59−4.66 Å and 4.57−4.73 Å for CBS9OBO9SCB and CBO9OBO9OCB, respectively, and both decreased with a reduction in temperature. As shown in Figure 6b, the *d*_WAX_ values were slightly smaller for thioether-linked CBS9OBO9SCB compared to the all-ether-linked CBO9OBO9OCB, indicating shorter lateral molecular correlations for the former. This tendency is similar to the behavior of thioether- and ether-containing LCs [65,66,68], and could be caused by two factors. The large dispersion forces provided by the more polarizable sulfur atom likely led to the shorter intermolecular lateral distances, while the electrostatic repulsion of electron-donating-ether-based LCs might lead to longer lateral distances [77].

Small-angle diffractions, symmetric on the meridional lines (Figure 6a and Figure S13), were observed at *2θ* = ~5.1° for CBS9OBO9SCB and CBO9OBO9OCB, indicating a strong cybotactic N (N_Cyb_) tendency with Sm-like clusters [78,79]. Such a pronounced N_Cyb_ trend is ascribable to the strong intermolecular interactions of LC trimers. The small-angle diffractions were not split on the meridional lines, indicating an N_Cyb_ nature consisting of orthogonal SmA-like clusters (namely, an N_CybA_ phase), similar to CBO*n*OBO*n*OCB (*n* = 10, 11) [58] and fluorenone-based trimer analogs [63]. This could be correlated to SmA phase formation for even-*n* trimer homologs. The *d*-spacing values of the small-angle diffractions (*d*_SAX_) of CBS9OBO9SCB and CBO9OBO9OCB were ~17 Å (Figure 6c), which could be approximately one-third of the average molecular lengths of both trimers. This indicates that this type of LC trimer forms N_CybA_ phases based on triply intercalated structures, irrespective of linkage type (thioether/ether) or spacer parity (even/odd) [58]. Strictly, the *d*_SAX_ values of all-ether-linked CBO9OBO9OCB seem like becoming gradually smaller than those of thioether-linked CBS9OBO9SCB with increasing Δ*T* (or decreasing *T*). 

The orientational order parameter (*S*) values of N phases were evaluated from wide-angle diffractions [6] and are plotted in Figure 6d as a function of Δ*T*. The *S* values were approximately 0.4–0.5 for CBS9OBO9SCB and 0.5–0.6 for CBO9OBO9OCB. These values, which are typical for N phases, gradually increased with increasing Δ*T* (or decreasing *T*). Over the entire N phase range, thioether-linked CBS9OBO9SCB exhibited lower *S* values than CBO9OBO9OCB (Figure 6d). This trend is similar to the difference between *bis*-thioether-linked CBS*n*SCB and mono-thioether-linked CBS*n*OCB dimers [68], indicating that replacement of ether with thioether leads to lower apparent S values. It is noted that apparent *S* values of bent LC dimers and oligomers based on wide-angle diffractions would not simply reflect orientational orders of the global molecular directors with rotational symmetry, which are strongly influenced by molecular biaxiality [3]. The thioether linkage enhances molecular biaxiality (or leads to more bending) due to the small C–S–C bond angle. Therefore, lower estimated apparent *S* values for thioether-linked dimers and oligomers mirror their more bent geometry in LC states than those of ether-linked analogs.

## 4. Conclusions

Herein, the development, phase-transition behavior, and mesophase structures of the homologous series of thioether-linked cyanobiphenyl-based trimers possessing a wide range of odd and even numbers of carbon atoms in the alkylene spacers, CBS*n*OBO*n*SCB (*n* = 3–11), have been reported for the first time. All CBS*n*OBO*n*SCB members exhibited LC phases. Odd–even oscillations in the phase-transition parameters were observed and were typically higher for even-*n* than for odd-*n*. Interestingly, even-*n* and odd-*n* homologs showed layered SmA and pseudo-layered heliconical N_TB_ phases, respectively. The differences in phase-transition behaviors, depending on the spacer parity of the LC trimers, were attributed to their different average molecular shapes or relative orientations between the biphenyls: A straightened stretched Z-like shape for even-*n* homologs and an obliquely directed bent shape for the odd-*n* homologs. Their phase-transition behavior and mesophase structures were compared with those of previously reported all-ether-linked CBO*n*OBO*n*OCB homologs. It was revealed that the replacement of outer ether linkages with thioether linkages primarily caused the LC trimers to form monotropic LCs and lower phase-transition temperatures. These phenomena were caused by the enhanced flexibility and bent shape (lower molecular anisotropy) of C–S–C bonds. The XRD analyses revealed that the LC trimers formed a cybotactic N_CybA_ phase with a triply intercalated structure, irrespective of the nature of the linkages (thioethers or ethers) or of spacer parity. This study provides new insights into the influence of thioether (C−S−C) bonds on the phase-transition properties of LC trimers.

## Figures and Tables

**Table 1:** Thermal phase sequences, phase-transition temperatures, and associated entropy changes (Δ*S*/*R*) for the CBS*n*OBO*n*SCB trimers. Upper and bottom lines correspond to heating data (*T*_m_, *T*_NI_, Δ*S*_m_/*R*, and Δ*S*_NI_/*R*) and cooling data (*T*_Cr_, *T*_NS_, *T*_NNTB_, *T*_IN_, Δ*S*_Cr_/*R*, and Δ*S*_IN_/*R*), respectively.

*n*	Cr	*T*_m_ (°C)	Δ*S*_m_/R	-	-	N	*T*_NI_ (°C)	Δ*S*_NI_/R	I
		*T*_Cr_ (°C)	Δ*S*_Cr_/R	Monotropic SmA or N_TB_^a^	*T*_NS_ or *T*_NNTB_ (°C)		*T*_IN_ (°C)	Δ*S*_IN_/R	
3	•	186.4	20.9						•
	•	144.8	19.6			•	142 ^a^	-	•
4	•	235.3	12.5			•	253.2	2.8	•
	•	196.6	11.9			•	249.5	3.1	•
5	•	171.8	22.5						•
	•	133.6	19.7	N_TB_	117 ^a^	•	154.2	0.4	•
6	•	219.0	24.7						•
	•	200.9	14.9	SmA	180 ^a^	•	217.4	4.0	•
7 ^b^	•	149.4	20.7			•	162.4	0.8	•
	•	130.0	20.6	N_TB_	126 ^a^	•	160.2	0.7	•
8	•	195.6	31.1 ^d^			•	198.8	- ^d^	•
	•	168.6	13.2	SmA	167 ^a^	•	195.0	4.3	•
9 ^c^	•	131.3	21.5			•	158.8	1.1	•
	•	103.6	20.3	N_TB_	122.7	•	156.5	1.1	•
10	•	184.8	32.8						•
	•	150.7	25.5	SmA	146 ^a^	•	176.2	4.9	•
11	•	135.4	29.2			•	154.0	1.7	•
	•	116.6	25.2	N_TB_	121 ^a^	•	151.5	1.7	•

^a^ Determined by POM. ^b^ Ref. [62] ^c^ Ref. [63] ^d^ Total entropy of (Δ*S*_m_/*R* + Δ*S*_NI_/*R*) due to peak overlap.

## Data Availability

Data are presented in the article and Supplementary Materials.

## Supplementary Materials

The following supporting information can be downloaded at: https://www.mdpi.com/article/10.3390/ma15051709/s1, Scheme S1: Synthesis pathway of odd-*n* CBS*n*OBO*n*SCB members; Scheme S2: Synthesis pathway of even-*n* CBS*n*OBO*n*SCB members; Figure S1: POM image of small N domains (140 °C) of CBS3OBO3SCB, developed from its supercooled Iso phase domains in a non-treated glass cell; Figure S2: POM image of a N phase at 140 °C of CBS4OBO4SCB in a non-treated glass cell; Figure S3: POM images of CBS5OBO5SCB, showing (a) a supercooled N domain at 121 °C, (b) a changed texture reminiscent of the N_TB_ phase at 171 °C, and (c) Cr phase at 105 °C in a non-treated glass cell; Figure S4: POM images of CBS8OBO8SCB showing (a) marble and schlieren textures (N phase) at 198 °C and (b) fan-shaped and focal conic textures (SmA phase) at 170 °C in a non-treated glass cell; Figure S5: POM image of fan-shaped and focal conic textures (SmA phase) at 133 °C of CBS10OBO10SCB in a non-treated glass cell; Figure S6: DSC curves of CBS3OBO3SCB; Figure S7: DSC curves of CBS4OBO4SCB; Figure S8: DSC curves of CBS5OBO5SCB; Figure S9: DSC curves of CBS6OBO6SCB; Figure S10: DSC curves of CBS8OBO8SCB; Figure S11: DSC curves of CBS10OBO10SCB; Figure S12: DSC curves of CBS11OBO11SCB; Figure S13: 2D-XRD pattern of the N phase 160 °C of CBO9OBO9OCB in a magnetic field denoted by a meridional arrow.
