# Supplementary material for: Thioether-Linked Liquid Crystal Trimers: Odd–Even Effects of Spacers and the Influence of Thioether Bonds on Phase Behavior
Source: Materials (Basel). 2022 Feb 24;15(5):1709. doi: 10.3390/ma15051709 (PMC8911043; doi:10.3390/ma15051709)
Supplement: Supplementary file 1 [file materials-15-01709-s001.zip › materials-1600225-supplementary.pdf]

materials

Article

Thioether-Linked Liquid Crystal Trimers: Odd–Even Effects of Spacers and the Influence of Thioether Bonds on Phase Behavior

Yuki Arakawa *, Kenta Komatsu, Yuko Ishida, Takuma Shiba and Hideto Tsuji

Department of Applied Chemistry and Life Science, Graduate School of Engineering, Toyohashi University of Technology, 1-1 Hibarigaoka, Tempaku-cho, Toyohashi, Aichi 441-8580, Japan; komatsu.kenta.qr@tut.jp (K.K.); ishida.yuko.rm@tut.jp (Y.I.); shiba.takuma.wt@tut.jp (T.S.); ht003@edu.tut.ac.jp (H.T.)

* Correspondence: arakawa@tut.jp

Citation: Arakawa, Y.; Komatsu, K.; Ishida, Y.; Shiba, T.; Tsuji, H. Thioether-Linked Liquid Crystal Trimers: Odd-Even Effects of Spacers and the Influence of Thioether Bonds on Phase Behavior. *Materials* **2022**, *15*, 1709. https://doi.org/10.3390/ma15051709

Academic Editor: Richard J. Mandle

Received: 31 January 2022
Accepted: 20 February 2022
Published: 24 February 2022

Publisher's Note: MDPI stays neutral with regard to jurisdictional claims in published maps and institutional affiliations.

Copyright: © 2022 by the authors. Licensee MDPI, Basel, Switzerland. This article is an open access article distributed under the terms and conditions of the Creative Commons Attribution (CC BY) license (https://creativecommons.org/licenses/by/4.0/).

1. Synthesis

All chemicals were commercially available and used as received. The odd CBS*n*OBO*n*SCB members were synthesized in a similar way reported previously for $n = 7$ and 9 as shown in Scheme 1 [1,2]. In this paper, the characterization data is presented for $n = 3, 5, 11$. The intermediates BrPhS*n*Br ($n = 3, 5, 6, 11$) were previously reported [3]. On the other hand, a different synthetic pathway was taken for even-*n* CBS*n*OBO*n*SCB members, as shown in Scheme S2, for the reason of their poor solubilities upon the course of the workup for purification.

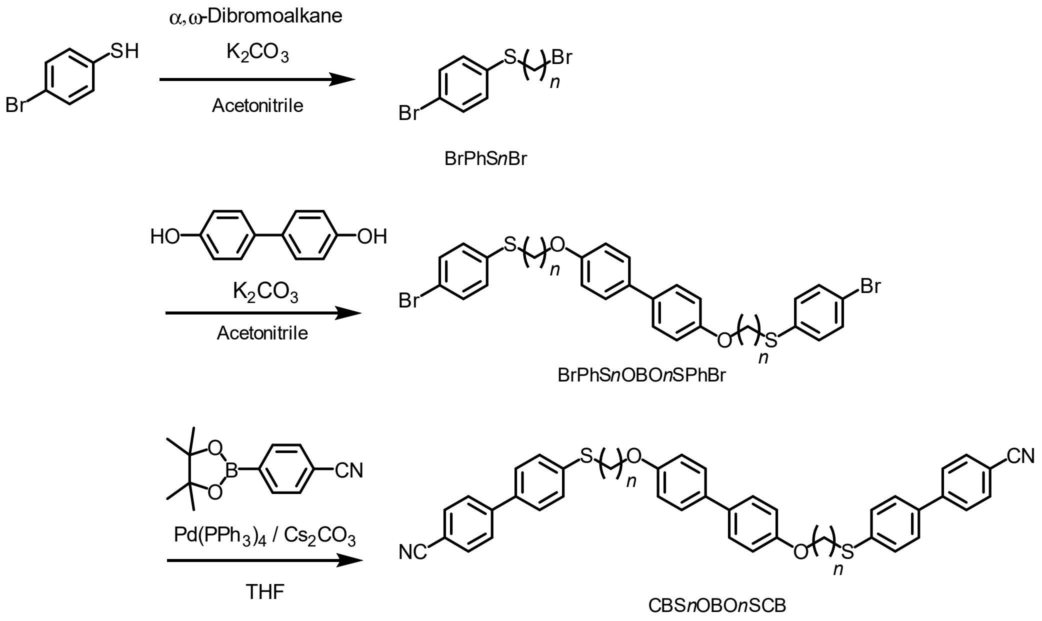

Scheme S1. Synthesis pathway of odd-*n* CBS*n*OBO*n*SCB members.

1.1. Synthesis of the Odd-n CBSnOBOnSCB Trimers

BrPhS3OBO3SPhBr (General procedures for odd-n BrPhS3OBO3SPhBr)

BrPhS3Br (0.220 g, 0.710 mmol), 4,4′-dihydroxybiphenyl (65.1 mg, 0.350 mmol), potassium carbonate (K_2CO_3) (0.236 g, 1.71 mmol), and acetonitrile (5 mL) were put in a double-neck flask and stirred at reflux temperature for 24 h. The mixture was extracted with dichloromethane and washed with water and brine. The separated organic phase was dried over magnesium sulfate ($MgSO_4$) and the volatiles were evaporated in vacuo. The residue was purified by column chromatography on silica gel [eluent: dichloromethane/hexane = 1/1

(v/v)] to afford a colorless solid. ^{1}H NMR (500 MHz, $CDCl_3$) δ 7.46 (d, 4H), 7.39 (d, 4H), 7.22 (d, 4H), 6.93 (d, 4H), 4.10 (t, 4H), 3.12 (t, 4H), 2.12 (tt, 4H) ppm. ^{13}C NMR (125 MHz, $CDCl_3$) δ 157.8, 135.5, 133.6, 131.9, 130.6, 127.7, 119.7, 114.7, 65.9, 30.2, 28.8 ppm.

BrPhS5OBO5SPhBr

^{1}H NMR (500 MHz, $CDCl_3$) δ 7.46 (d, 4H), 7.38 (d, 4H), 7.18 (d, 4H), 6.92 (d, 4H), 3.98 (t, 4H), 2.93 (t, 4H), 1.81 (tt, 4H), 1.72 (tt, 4H), 1.62 (tt, 4H) ppm. ^{13}C NMR (125 MHz, $CDCl_3$) δ 158.0, 135.9, 133.4, 131.9, 130.5, 127.7, 119.5, 114.7, 67.6, 35.6, 28.8, 28.7, 25.3ppm.

BrPhS11OBO11SPhBr

^{1}H NMR (500 MHz, $CDCl_3$) δ 7.46 (d, 4H), 7.38 (d, 4H), 7.17 (d, 4H), 6.94 (d, 4H), 3.98 (t, 4H), 2.89 (t, 4H), 1.79 (tt, 4H), 1.63 (tt, 4H), 1.51–1.20 (m, 28H) ppm. ^{13}C NMR (125 MHz, $CDCl_3$) δ 158.2, 136.2, 133.3, 131.8, 130.3, 127.6, 119.3, 114.7, 68.0, 33.6, 29.51, 29.45, 29.43, 29.36, 29.3, 29.1, 29.0, 28.8, 26.0 ppm.

CBS3OBO3SCB (General procedures for odd-n CBSnOBOnSCB)

4-Cyanophenylboronic acid pinacol ester (0.150 g, 0.655 mmol), BrPhS3OBO3SPhBr (0.206 g, 0.320 mmol), Cs_2CO_3 (0.432 g, 1.32 mmol), and $Pd(PPh_3)_4$ (93.6 mg, 81.0 μmol) were put in a double-neck flask purged with an argon gas. Subsequently, tetrahydrofurane (THF) (5 mL) degassed by bubbling an argon gas was added to the prior flask, and the mixture was stirred at reflux temperature for 24 h. The mixture was extracted with dichloromethane, washed with water and brine, and the separated organic layer was dried over $MgSO_4$. After the volatiles were removed in vacuo, the residue was purified by silica gel column chromatography [eluent: dichloromethane/hexane = 5/1 (v/v) to dichloromethane] and recrystallised in dichloromethane, to afford a colorless solid. ^{1}H NMR (500 MHz, $CDCl_3$) δ 7.68 (d, 4H), 7.62 (d, 4H), 7.47 (d, 4H), 7.43 (d, 4H), 7.43 (d, 4H), 6.92 (d, 4H), 4.13 (t, 4H), 3.22 (t, 4H), 2.19 (tt, 4H) ppm. ^{13}C NMR (125 MHz, $CDCl_3$) δ 157.8, 144.8, 137.7, 136.4, 133.4, 132.6, 128.9, 127.63, 127.56, 127.3, 118.9, 114.7, 110.8, 65.8, 28.6, 29.0 ppm.

CBS5OBO5SCB

This compound was synthesized in a procedure similar to CBS3OBO3SCB. ^{1}H NMR (500 MHz, $CDCl_3$) δ 7.69 (d, 4H), 7.64 (d, 4H), 7.47 (d, 4H), 7.44 (d, 4H), 7.39 (d, 4H), 6.92 (d, 4H), 4.00 (t, 4H), 3.02 (t, 4H), 1.85 (tt, 4H), 1.78 (tt, 4H), 1.72–1.62 (m, 4H) ppm. ^{13}C NMR (125 MHz, $CDCl_3$) δ 158.1, 144.9, 138.2, 136.2, 133.3, 132.6, 128.8, 127.6, 127.5, 127.3, 118.9, 114.7, 110.7, 67.6, 33.0, 28.8, 28.7, 25.3 ppm.

CBS11OBO11SCB

^{1}H NMR (500 MHz, $CDCl_3$) δ 7.71 (d, 4H), 7.66 (d, 4H), 7.50 (d, 4H), 7.45 (d, 4H), 7.38 (d, 4H), 6.93 (d, 4H), 3.98 (t, 4H), 2.97 (t, 4H), 1.79 (tt, 4H), 1.69 (tt, 4H), 1.54–1.40 (m, 8H), 1.40–1.23 (m, 20H) ppm. ^{13}C NMR (125 MHz, $CDCl_3$) δ 158.2, 144.9, 138.6, 136.0, 133.3, 132.6, 128.5, 127.6, 127.5, 127.3, 119.0, 114.7, 110.7, 68.0, 33.0, 29.5, 29.45, 29.45, 29.4, 29.3, 29.1, 29.0, 28.8, 26.0 ppm.

1.2. Synthesis of the Even-n CBSnOBOnSCB Trimers

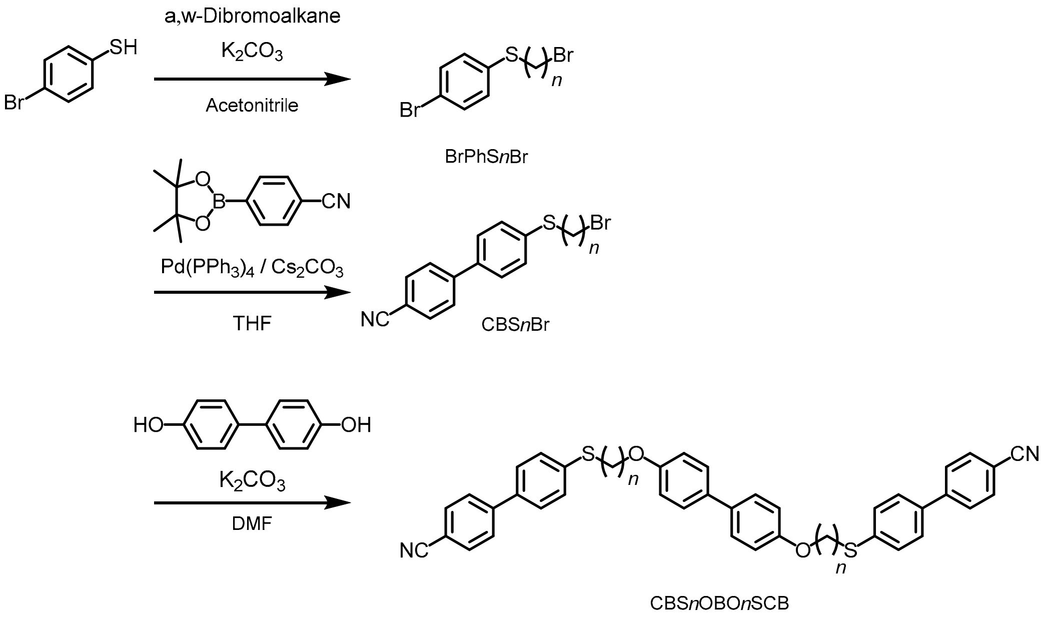

Scheme S2. Synthesis pathway of even-*n* CBS*n*OBO*n*SCB members.

BrPhS4Br (Gereral procedure for even-n BrPhSnBr)

4-Bromobenzenethiol (0.501 g, 2.65 mmol), 1,4-dibromobutane (0.883, 4.09 mmol), K_2CO_3 (0.729 g, 5.27 mmol) and acetonitrile (50 mL) were put in a flask and the mixture was stirred at ambient temperature. After 2h, the reaction mixture was extracted with dichloromethane, washed with water and brine, and dried over $MgSO_4$. After removing the volatiles in vacuo, the residue was purified by column chromatography on silica gel [eluent: dichloromethane/hexane = 1/5 (v/v)], to afford a colorless solid. ^{1}H NMR (500 MHz, $CDCl_3$) δ 7.40 (d, 2H), 7.19 (d, 2H), 3.41 (t, 2H), 2.92 (t, 2H), 2.00 (tt, 2H), 1.79 (tt, 2H) ppm. ^{13}C NMR (125 MHz, $CDCl_3$) δ 135.5, 131.9, 130.8, 119.8, 32.9, 32.9, 31.4, 27.4 ppm.

BrPhS6Br

^{1}H NMR (500 MHz, $CDCl_3$) δ 7.39 (d, 2H), 7.18 (d, 2H), 3.40 (t, 2H), 2.90 (t, 2H), 1.86 (tt, 2H), 1.65 (tt, 2H), 1.45 (tt, 4H) ppm. ^{13}C NMR (125 MHz, $CDCl_3$) δ ppm. ^{13}C NMR (125 MHz, $CDCl_3$) 136.0, 131.8, 130.4, 119.5, 33.8, 33.5, 32.5, 28.7, 27.8, 27.6 ppm.

BrPhS8Br

^{1}H NMR (500 MHz, $CDCl_3$) δ 7.39 (d, 2H), 7.17 (d, 2H), 3.40 (t, 2H), 2.89 (t, 2H), 1.85 (tt, 2H), 1.63 (tt, 2H), 1.42 (tt, 4H), 1.35–1.27 (m, 4H) ppm. ^{13}C NMR (100 MHz, $CDCl_3$) δ 136.2, 131.8, 130.3, 119.4, 34.0, 33.6, 32.7, 28.91, 28.88, 28.60, 28.56, 28.0 ppm.

BrPhS10Br

^{1}H NMR (500 MHz, $CDCl_3$) δ 7.39 (d, 2H), 7.17 (d, 2H), 3.41 (t, 2H), 2.89 (t, 2H), 1.85 (tt, 2H), 1.62 (tt, 2H), 1.46–1.36 (m, 4H), 1.34–1.23 (m, 8H) ppm. ^{13}C NMR (125 MHz, $CDCl_3$) δ 136.3, 131.8, 130.3, 119.3, 34.0, 33.6, 32.8, 29.3, 29.3, 29.1, 28.9, 28.72, 28.7, 28.1 ppm.

Synthesis of CBS4Br (General procedure for even-n CBS4Br members)
BrPhS4Br (0.350 g, 1.08 mmol), 4-Cyanophenylboronic acid pinacol ester (0.266 g, 1.16 mmol), Cs_2CO_3 (0.704 g, 2.16 mmol), and $Pd(PPh_3)_4$ (0.159 g, 0.138 mmol) were put in a double-neck flask purged with an argon gas. Subsequently, tetrahydrofurane (THF) (5 mL) degassed by bubbling an argon gas was added to the prior flask, and the mixture was stirred at reflux temperature for 24 h. The mixture was extracted with dichloromethane, washed with water and brine, and the separated organic layer was dried over $MgSO_4$. After the volatiles were removed in vacuo, the residue was purified by silica gel column chromatography [eluent: dichloromethane/hexane = 5/1 (v/v)]. ^{1}H NMR (500 MHz, $CDCl_3$) δ 7.72 (d, 2H), 7.66 (d, 2H), 7.52 (d, 2H), 7.41 (d, 2H), 3.43 (t, 2H), 3.00 (t, 2H), 2.00 (tt, 2H), 1.86 (tt, 2H) ppm. ^{13}C NMR (125 MHz, $CDCl_3$) δ 144.8, 137.7, 136.5, 132.6, 129.0, 127.6, 127.3, 118.9, 110.8, 32.9, 32.3, 31.5, 27.4 ppm.

CBS6Br
^{1}H NMR (400 MHz, $CDCl_3$) δ 7.72 (d, 2H), 7.66 (d, 2H), 7.51 (d, 2H), 7.39 (d, 2H), 3.41 (t, 2H), 2.98 (t, 2H), 1.87 (tt, 2H), 1.71 (tt, 2H), 1.53–1.44 (m, 4H) ppm. ^{13}C NMR (100 MHz, $CDCl_3$) 144.9, 138.2, 136.1, 32.6, 128.6, 127.5, 127.3, 118.9, 110.7, 33.8, 32.9, 32.5, 28.7, 27.9, 27.6 ppm.

CBS8Br
^{1}H NMR (500 MHz, $CDCl_3$) δ 7.74 (d, 2H), 7.66 (d, 2H), 7.51 (d, 2H), 7.39 (d, 2H), 3.40 (t, 2H), 2.97 (t, 2H), 1.85 (tt, 2H), 1.69 (tt, 2H), 1.44 (tt, 4H), 1.38–1.28 (m, 4H) ppm. ^{13}C NMR (125 MHz, $CDCl_3$) δ 144.9, 138.5, 136.1, 132.6, 128.6, 127.5, 127.3, 118.9, 110.7, 34.0, 33.0, 32.7, 28.9, 28.9, 28.7, 28.6, 28.0 ppm.

CBS10Br
^{1}H NMR (500 MHz, $CDCl_3$) δ 7.71 (d, 2H), 7.66 (d, 2H), 7.51 (d, 2H), 7.39 (d, 2H), 3.40 (t, 2H), 2.97 (t, 2H), 1.85 (tt, 2H), 1.69 (tt, 2H), 1.43 (tt, 4H), 1.36–1.23 (m, 8H) ppm. ^{13}C NMR (125 MHz, $CDCl_3$) δ 144.9, 138.6, 136.0, 132.6, 128.5, 127.5, 127.3, 118.9, 110.7, 34.0, 33.0, 32.8, 29.33, 29.30, 29.1, 29.0, 28.8, 28.7, 28.1 ppm.

CBS4OBO4SCB (General procedure for even-n CBSnOBOnSCB members)
A mixture of CBS4Br (0.121 g, 0.349 mmol), 4,4'-dihydroxybiphenyl (32.5 mg, 0.175 mmol), potassium carbonate (96.5 mg, 0.698 mmol) and DMF (2 mL) was stirred at reflux temperature overnight. the mixture was cooled to room temperature, and filtrated. The residue was washed with plenty of distilled water, and dried *in vacuo*. A small amoung of the residue was taken and purified by column chromatography on silica gel (eluent: chloroform) and recrystallization from chloroform to afford a colorless solid. ^{1}H NMR (500 MHz, $CDCl_3$) δ 7.71 (d, 4H), 7.65 (d, 4H), 7.50 (d, 4H), 7.45 (d, 4H), 7.41 (d, 4H), 6.93 (d, 4H), 4.03 (t, 4H), 3.07 (t, 4H), 1.98 (tt, 4H), 1.91 (tt, 4H) ppm.

CBS6OBO6SCB
^{1}H NMR (500 MHz, $CDCl_3$) δ 7.70 (d, 4H), 7.65 (d, 4H), 7.50 (d, 4H), 7.44 (d, 4H), 7.39 (d, 4H), 6.93 (d, 4H), 3.99 (t, 4H), 3.00 (t, 4H), 1.81 (tt, 4H), 1.74 (tt, 4H), 1.58–1.48 (m 8H) ppm.

CBS8OBO8SCB
^{1}H NMR (500 MHz, $CDCl_3$) δ 7.71 (d, 4H), 7.66 (d, 4H), 7.51 (d, 4H), 7.45 (d, 4H), 7.39 (d, 4H), 6.93 (d, 4H), 3.98 (t, 4H), 2.98 (t, 4H), 1.79 (tt, 4H), 1.70 (tt, 4H), 1.55–1.38 (m 16H) ppm.

CBS10OBO10SCB

^{1}H NMR (500 MHz, $CDCl_3$) δ 7.71 (d, 4H), 7.66 (d, 4H), 7.50 (d, 4H), 7.45 (d, 4H), 7.39 (d, 4H), 6.93 (d, 4H), 3.98 (t, 4H), 2.97 (t, 4H), 1.79 (tt, 4H), 1.69 (tt, 4H), 1.50–1.41 (m, 8H), 1.39–1.29 (m, 16H) ppm.

2. POM images

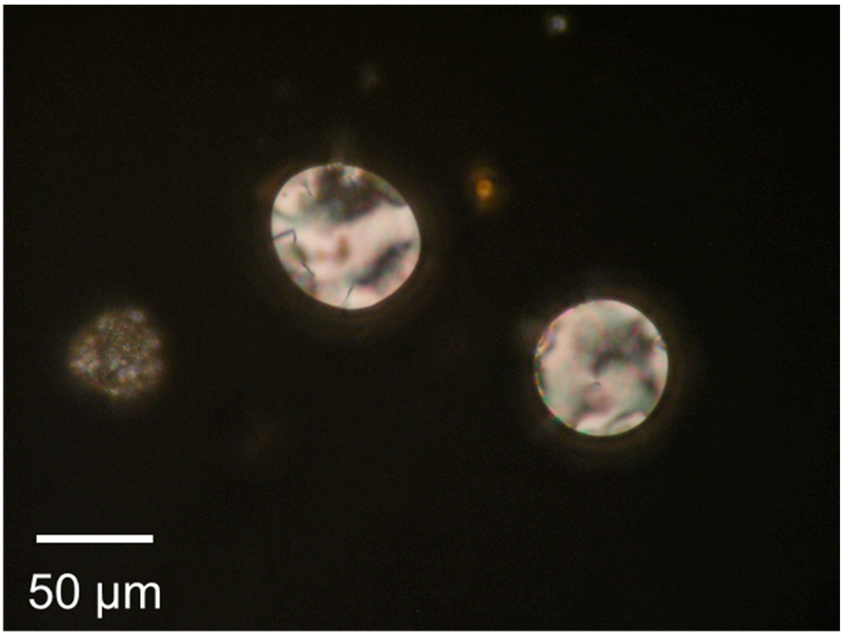

Figure S1. POM image of small N domains (140 °C) of CBS3OBO3SCB, developed from its supercooled Iso phase domains in a non-treated glass cell.

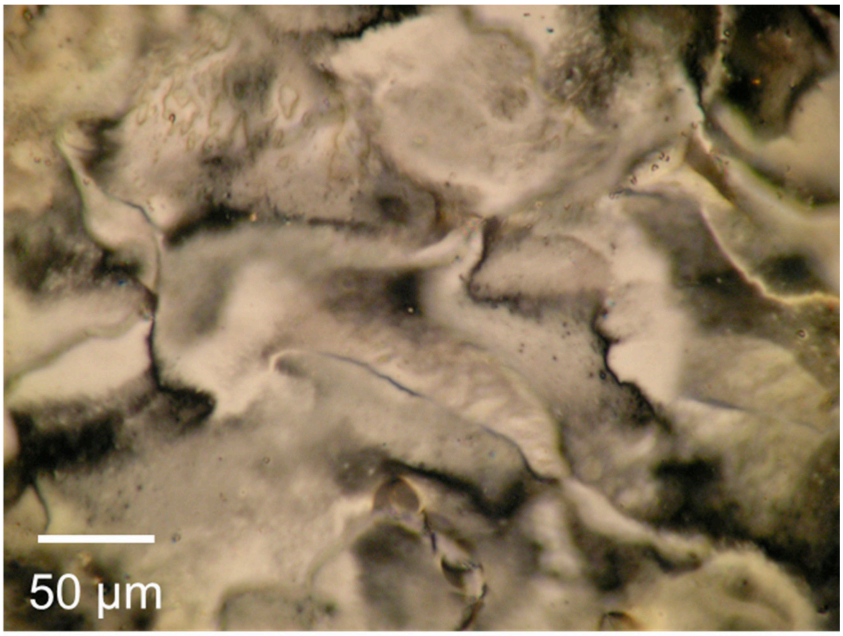

Figure S2. POM image of a N phase at 140 °C of CBS4OBO4SCB in a non-treated glass cell.

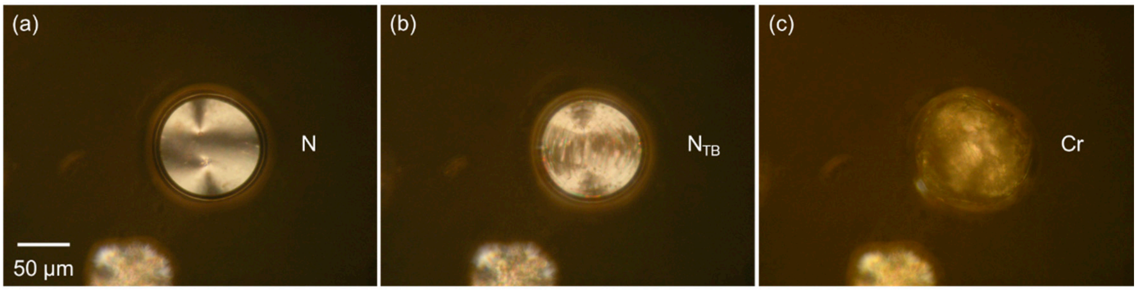

Figure S3. POM images of CBS5OBO5SCB, showing (**a**) a supercooled N domain at 121 °C, (**b**) a changed texture reminiscent of the N_{TB} phase at 171 °C, and (**c**) Cr phase at 105 °C in a non-treated glass cell.

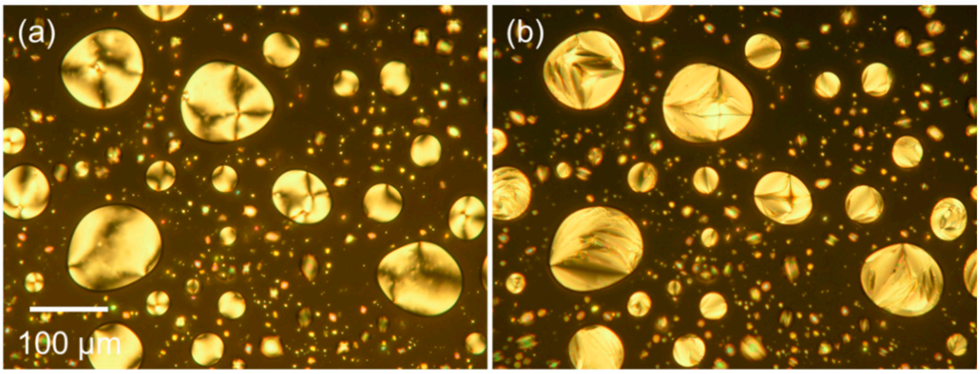

Figure S4. POM images of CBS8OBO8SCB showing (**a**) marble and schlieren textures (N phase) at 198 °C and (**b**) fan-shaped and focal conic textures (SmA phase) at 170 °C in a non-treated glass cell.

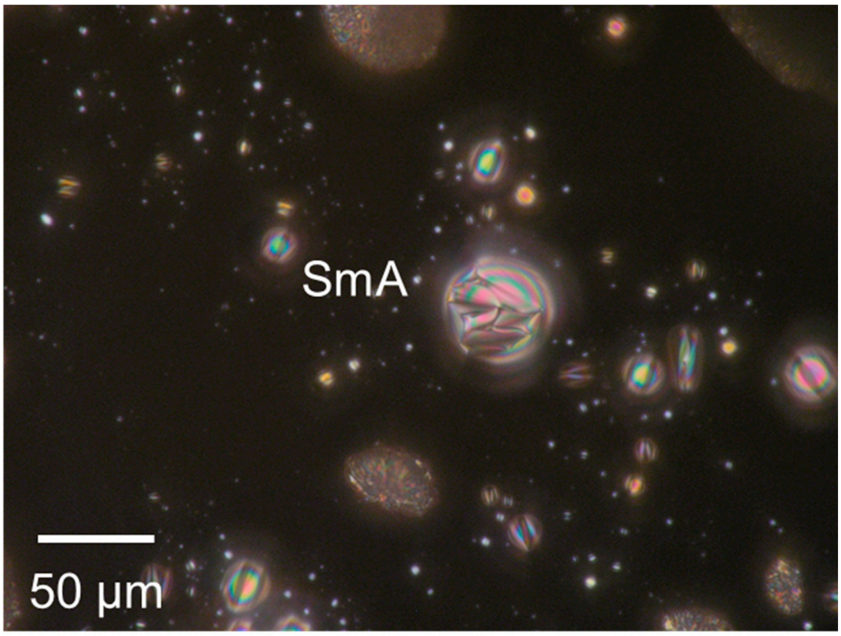

Figure S5. POM image of fan-shaped and focal conic textures (SmA phase) at 133 °C of CBS10OBO10SCB in a non-treated glass cell.

3. DSC

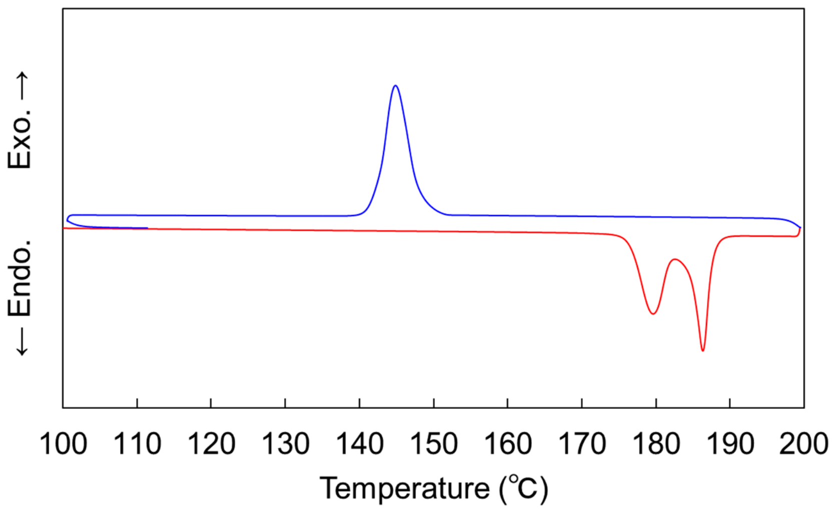

Figure S6. DSC curves of CBS3OBO3SCB.

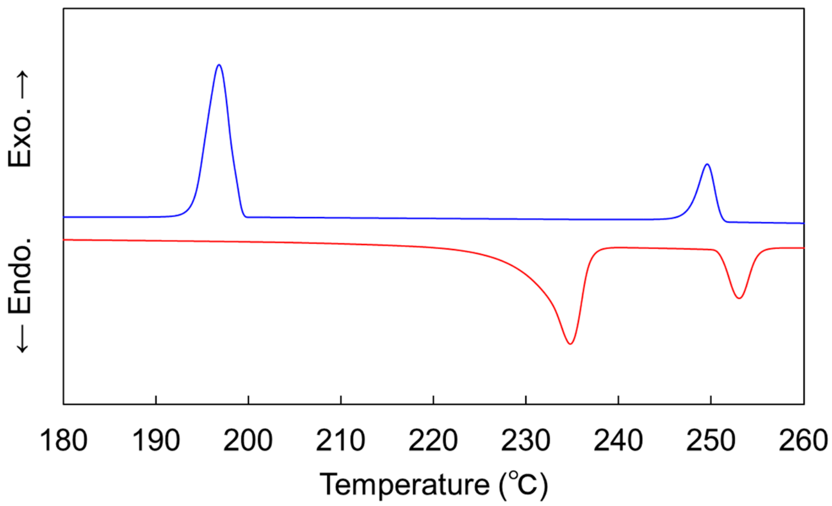

Figure S7. DSC curves of CBS4OBO4SCB.

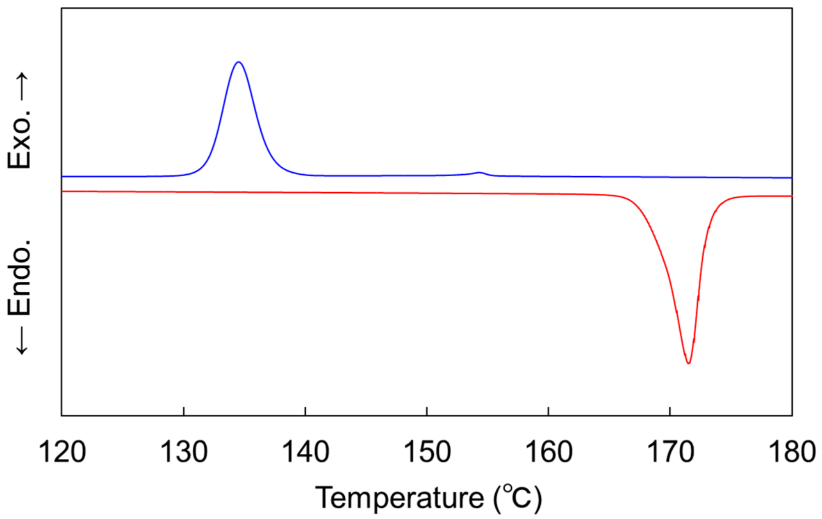

Figure S8. DSC curves of CBS5OBO5SCB.

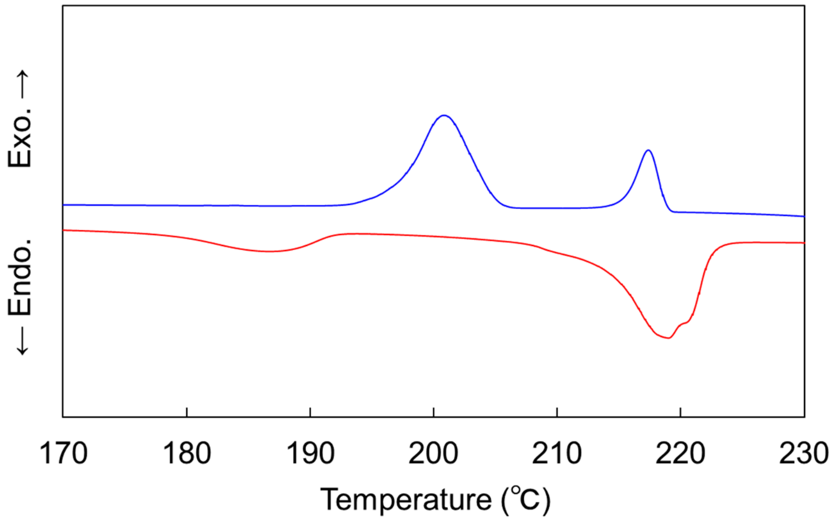

Figure S9. DSC curves of CBS6OBO6SCB.

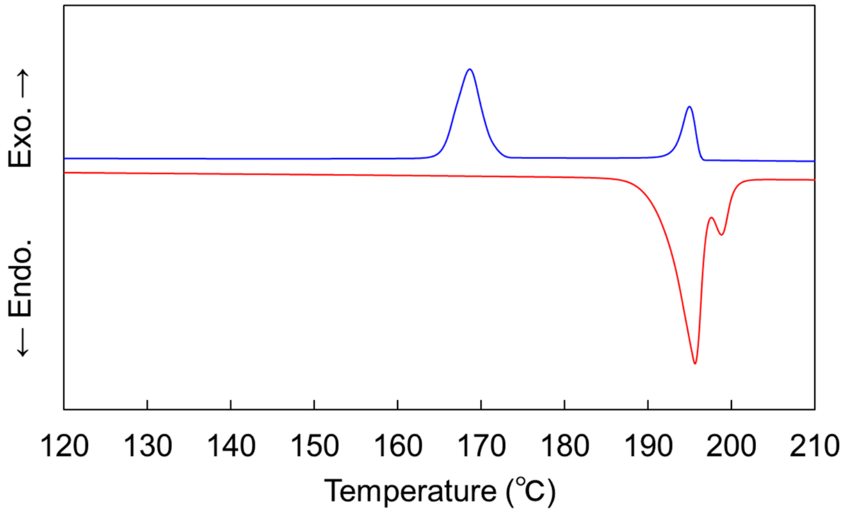

Figure S10. DSC curves of CBS8OBO8SCB.

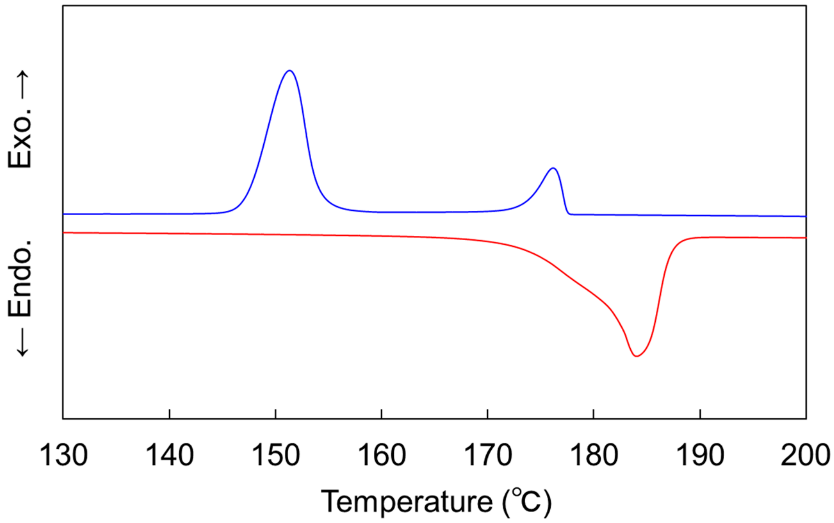

Figure S11. DSC curves of CBS10OBO10SCB.

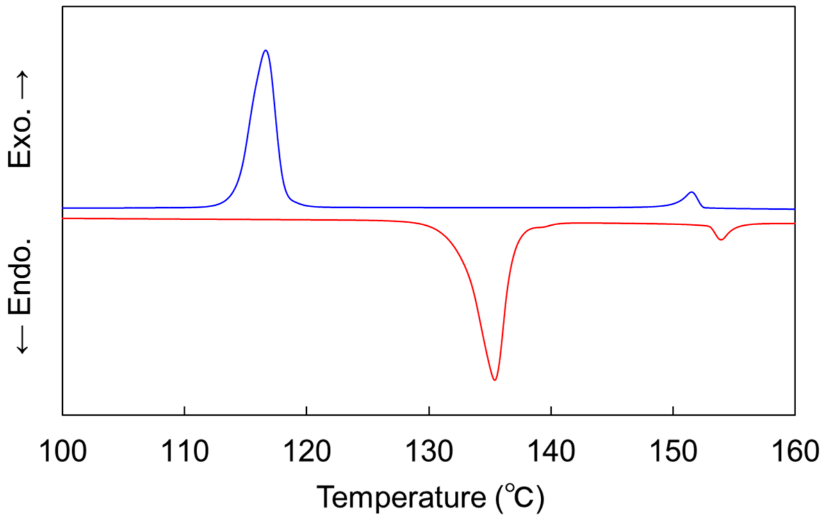

Figure S12. DSC curves of CBS11OBO11SCB.

4. XRD

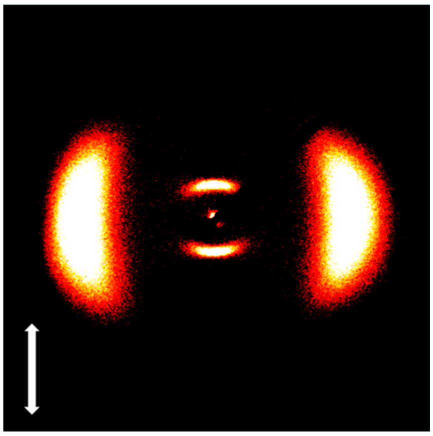

Figure S13. The 2D-XRD pattern of the N phase 160 °C of CBO9OBO9OCB in a magnetic field denoted by a meridional arrow.

References

1. Arakawa, Y.; Komatsu, K.; Inui, S.; Tsuji, H. Thioether-linked liquid crystal dimers and trimers: The twist-bend nematic phase. *J. Mol. Struct.* **2020**, *1199*, 126913.
2. Arakawa, Y.; Komatsu, K.; Tsuji, H. 2,7-Substituted fluorenone-based liquid crystal trimers: Twist-bend nematic phase induced by outer thioether linkage. **2022**, https://doi.org/10.1080/01411594.2022.2044040.
3. Arakawa, Y.; Ishida, Y.; Komatsu, K.; Arai, Y.; Tsuji, H. Thioether-linked benzylideneaniline-based twist-bend nematic liquid crystal dimers: Insights into spacer lengths, mesogenic arm structures, and linkage types. *Tetrahedron* **2021**, *95*, 132351.
